# The screening of pivotal gene expression signatures and biomarkers in renal carcinoma

**DOI:** 10.7150/jca.30656

**Published:** 2019-10-19

**Authors:** Hailong Ruan, Sen Li, Junwei Tong, Qi Cao, Zhengshuai Song, Keshan Wang, Yu huang, Lin Bao, Xuanyu Chen, Hongmei Yang, Ke Chen, Xiaoping Zhang

**Affiliations:** 1 Department of Urology, Union Hospital, Tongji Medical College, Huazhong University of Science and Technology, Wuhan 430022, China; 2 Institute of Urology, Union Hospital, Tongji Medical College, Huazhong University of Science and Technology, Wuhan 430022, China; 3 Department of Pathogenic Biology, School of Basic Medicine, Huazhong University of Science and Technology, Wuhan 430030, China

**Keywords:** hub genes, clear cell renal cell carcinoma, biomarkers, bioinformatics, differentially expressed gene

## Abstract

Renal cell carcinoma (RCC) is one of the most common malignancies in the urinary system, among which the proportion of clear cell RCC (ccRCC) is over 80%. This study aims to explore potential signaling pathways and key biomarkers that drive RCC progression. The RCC GEO Datasets GSE15641 was featured to screen differentially expressed genes (DEGs). The pathway enrichment and functional annotation of differentially expressed genes were analyzed using the Kyoto Encyclopedia of Genes and Genomes (KEGG) and the Gene Ontology (GO). We screened Hub genes from DEGs using protein-protein interaction (PPI) networks and Cytoscape software. The survival and diagnostic analysis of these hub genes was performed to evaluate their potential prognostic and diagnostic value for ccRCC. In GSE15641 dataset, 598 DEGs were captured according to screening criteria (406 up-regulated genes and 192 down-regulated genes). Meanwhile, 15 hub genes were screened out from DEGs using PPI and Cytoscape. Kaplan Meier and ROC curve analysis identified three potential prognostic and diagnostic biomarkers (TGFB1, TIMP1 and VIM) for ccRCC from 15 hub genes. Gene set enrichment analysis (GSEA) revealed that these three dysregulated genes are mainly enriched in primary immunodeficiency, ECM receptor interaction, cytokine receptor interaction and natural killer cell-mediated cytotoxicity pathway. In summary, our findings discovered pivotal gene expression signatures and signaling pathways in the progression of ccRCC. TGFB1, TIMP1 and VIM might contribute to the progression of ccRCC, which could have potential as biomarkers or therapeutic targets for ccRCC.

## Introduction

Renal cell carcinoma (RCC) is one of the most common malignant tumors in the genitourinary tract. RCC can be divided into many pathological subtypes, among which clear cell RCC (ccRCC) is the most common subtype. Current major treatments for ccRCC include surgical resection, targeted drug therapy and immunotherapy. Although, in recent years, surgical techniques have been continuously improved and targeted drugs have been used clinically, the prognosis of some patients with advanced or metastatic ccRCC is still not ideal 1. Moreover, ccRCC is not sensitive to traditional chemoradiotherapy and possesses the risk of high metastasis and high mortality, resulting in poor prognosis 2. Therefore, early diagnosis and early treatment are the key steps to improve the cure rate of ccRCC. However, early accurate diagnosis of ccRCC remains a great challenge for clinicians 3, and there is a lack of diagnostic biomarkers at this stage.

Mutation and abnormal expression of genes contribute to the initiation and progression of tumors. Mastering the changes in the characteristics of key genes facilitates a comprehensive understanding of ccRCC progression and screening for molecular markers. Numerous studies have confirmed that VHL mutations are key drivers of ccRCC initiation and development 4-6, and that VHL mutations can serve as prognostic markers for ccRCC 7. A separate VHL mutation is not enough to cause ccRCC, as the formation of ccRCC is a complex biological process involving multiple genes. Previous studies have identified that mutations and abnormal expression of PBRM1 8, TCEB1 9 and PP5 10 contribute to the progression of ccRCC. Comprehensive insight into genetic characteristics of ccRCC could help us discover novel diagnostic biomarkers and therapeutic targets for precise treatment of ccRCC patients.

The wide application of high-throughput gene microarray expression profiling for exploring global genomes molecular landscape could help us easily find differentially expressed genes (DEGs) from cancerous and para-cancerous tissues. However, the application of microarray expression profiles in clinical practice is largely limited, because thousands of genes are identified by microarray expression profiles, resulting in lack of relevant experimental validation and statistical analysis 11. To apply these differentially expressed genes to clinical practice as soon as possible, it is necessary to screen out a suitable number of core genes from so many DEGs.

In this study, we downloaded GSE15641 dataset from the Gene Expression Omnibus (GEO) and comprehensively screened out DEGs by using GEO2R online tools. Subsequently, we constructed a protein-protein interaction network of DEGs and sieved the top15 hub gene with a high degree of connectivity through Cytoscape tool. Moreover, we performed the Kyoto Encyclopedia of Genes and Genomes (KEGG) and the Gene Ontology (GO) analysis of DEGs and found key biological features and signaling pathways. Overall survival and disease-free survival analysis of 15 hub genes were used to evaluate their prognostic value. The high expression of three genes, (TGFB1, TIMP1 and VIM) predicted a worse prognosis. Analysis of receiver operating characteristic (ROC) curves revealed that these three genes have potential as diagnostic markers for ccRCC. Finally, we used The Cancer Genome Atlas (TCGA) and the Human Protein Atlas (http://www.proteinatlas.org) database to verify the expression of mRNA and protein levels of these three genes in cancerous and para-cancerous tissues.

## Materials and Methods

### Data collection

We downloaded the GSE15641 dataset from the GEO database, which is a publicly available cancer database. The microarray data of GSE15641 dataset include 23 normal kidney tissues and 32 ccRCC tissues, which was based on GPL96 platform ([HG-U133A] Affymetrix Human Genome U133A Array) by Jones et al 12.

### Data processing

The differentially expressed genes between 32 ccRCC tissues and 23 normal kidney tissues were screened out using GEO2R (https://www.ncbi.nlm.nih.gov/geo/geo2r/), a free online analysis software for the GEO database, which was dependent on R language programming. According to the screening criteria of logFC≥2 or logFC≤-2 and adjusted P value <0.05, 598 DEGs were screened out, of which 406 genes were up-regulated and 192 genes were down-regulated. Then, we used Cytoscape software to select the top 15 genes as hub genes based on a high degree of connectivity.

In addition, we performed a heat map of top 50 DEGs using GraphPad Prism 7.04 and a volcano plot of all genes using ImageGP (http://www.ehbio.com/ImageGP) after downloading the raw data of the GSE15641 dataset.

### KEGG signaling pathway enrichment and GO functional annotation analysis

The Kyoto Encyclopedia of Genes and Genomes (KEGG) and the Gene Ontology (GO) analysis of DEGs were performed using The Database for Annotation, Visualization and Integrated Discovery (https://david.ncifcrf.gov/) (DAVID, version 6.8), which provides a comprehensive set of functional annotation tools for investigators to understand biological meaning behind large list of genes.

### PPI network construction and screening of hub gene

The protein-protein interaction (PPI) networks of DEGs were constructed using The Search Tool for the Retrieval of Interacting Genes (STRING), which is a publicly available software for assessing the interaction between proteins and proteins (https://string-db.org/). Cytoscape software was used to visualize the PPI network and select the top 15 gene as the hub genes.

### Kaplan Meier analysis of Hub genes

The overall survival (OS) analysis of hub genes was performed using the online Oncolnc survival analysis server (http://www.oncolnc.org/). The disease-free survival (DFS) analysis of hub genes was performed using the online GEPIA survival analysis server (http://gepia.cancer-pku.cn/). The logrank P value was calculated and analyzed, and we screened out three promising hub genes ((TGFB1, TIMP1 and VIM)) with the prognostic value.

### Verification of the expression level of hub genes

We downloaded the mRNA expression and TNM stage parameters of three hub genes from the TCGA database and compared their expression between ccRCC tissues and normal kidney tissues. Subsequently, we analyzed the association of mRNA expression levels of the three hub genes with TNM stage. The protein expression levels of the three hub genes were evaluated using The Human Protein Atlas (HPA, https://www.proteinatlas.org/).

### ROC curves analysis of hub genes

The potential diagnostic value of the three hub genes was evaluated using the receiver operating characteristic (ROC) curves by Graphpad Prism software.

### Gene set enrichment analysis of hub genes

After downloading the mRNA levels of the three hub genes from TCGA, they were divided into high expression group and low expression group according to the median value, and then GSEA analysis was performed.

### Statistical Analysis

The values of different groups are represented by the mean ± SD. The comparison of the expression levels of the paired tumor tissues was analyzed by a paired t test. The comparison of expression levels of unpaired tumor tissues was analyzed by unpaired t test. P < 0.05 was considered statistically significant.

## Results

### The screening of DEGs and Hub Genes in ccRCC

**Figure 1:**
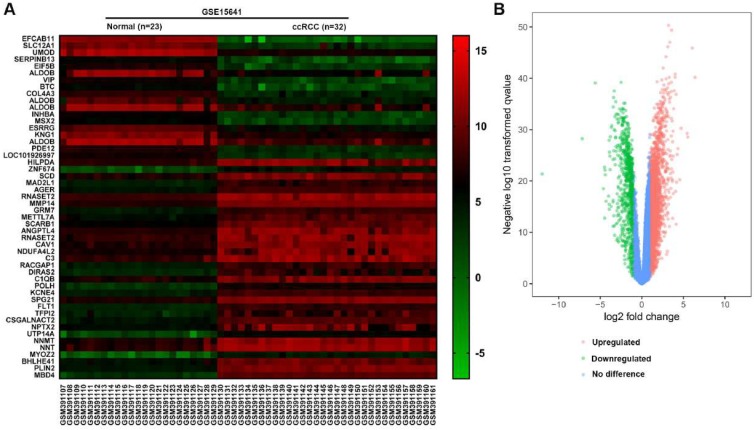
(A) The heat map of top 50 DEGs. (B) The volcano map of genes in ccRCC from GSE15641 dataset. Red dots represent the upregulated DEGs, green dots represent the downregulated DEGs, and blue dots represent the genes with no difference in expression.

In the GSE15641 dataset, there were 23 normal renal tissues and 32 ccRCC tissues. According to the screening criteria of logFC≥2 or logFC≤-2 and adjusted P value <0.05, 598 DEGs were screened out by the GEO2R analysis tool, of which 406 genes were up-regulated and 192 genes were down-regulated (Fig. 1B). According to the value of **|**log FC**|**, we picked out the top 50 genes that differentially expressed in ccRCC. The top 50 DEGs were presented in the form of a heat map. Furthermore, 15 Hub genes with a higher connectivity degree were screened out from the GSE15641 dataset (Table 1).

### KEGG signaling pathway enrichment and GO functional annotation analysis of DEGs in ccRCC

**Figure 2:**
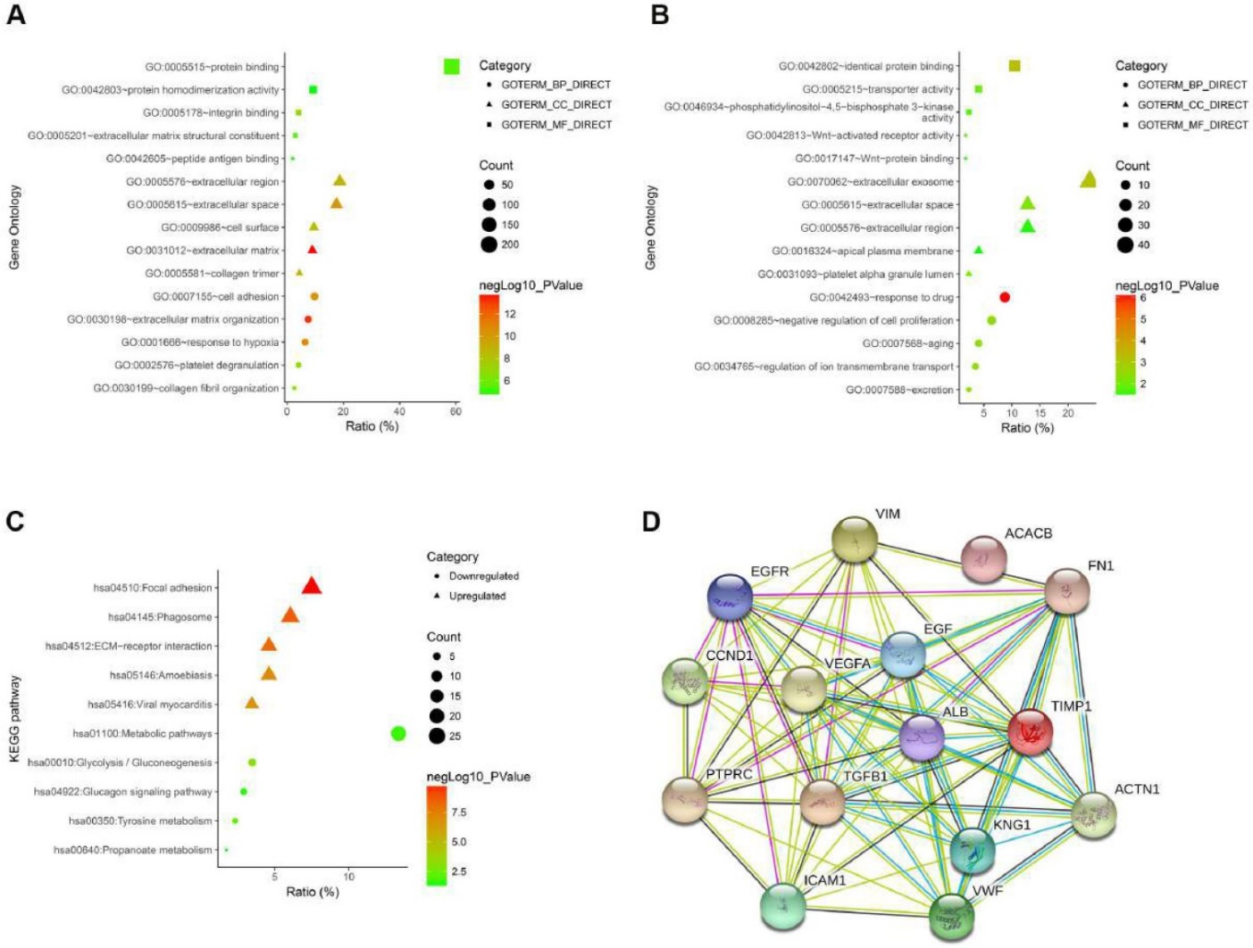
(A) The GO analysis of upregulated DEGs. (B) The GO analysis of downregulated DEGs. (C) The KEGG pathway analysis of DEGs. (D) The PPI network of top 15 hub genes.

To gain an in-depth and comprehensive understanding of the biological characteristics of these DEGs, GO functional annotation and KEGG signaling pathway enrichment analysis were performed through online analytical tool DAVID. GO analysis showed that the enriched biological processes (BP) of up-regulated DEGs include extracellular matrix organization, response to hypoxia, cell adhesion, et al. The enriched molecular function (MF) of up-regulated DEGs involved integrin binding, protein binding, extracellular matrix structural constituent, et al. The involved cellular components (CC) of up-regulated DEGs included extracellular matrix, extracellular space, collagen trimer, et al (Fig. 2A and Table 2). GO analysis showed that the enriched biological processes (BP) of down-regulated DEGs include response to drug, negative regulation of cell proliferation, regulation of ion transmembrane transport, et al. The enriched molecular function (MF) of down-regulated DEGs involved Wnt-activated receptor activity, phosphatidylinositol-4,5-bisphosphate 3-kinase activity, Wnt-protein binding, et al. The involved cellular components (CC) of down-regulated DEGs included extracellular exosome, platelet alpha granule lumen, apical plasma membrane, et al (Fig. 2B and Table 2). As shown in Figure 2C, we performed the KEGG pathway enrichment analysis of DEGs. KEGG analysis revealed that the enriched pathways of up-regulated DEGs include Focal adhesion, ECM-receptor interaction, viral myocarditis, while the down-regulated DEGs were enriched in tyrosine metabolism, glucagon signaling pathway, metabolic pathways (Fig. 2C and Table 3). Finally, we performed a PPI network of the top 15 hub genes using the STRING online analysis tool (Fig. 2D).

### The prognostic value assessment of the top 15 Hub genes

**Figure 3:**
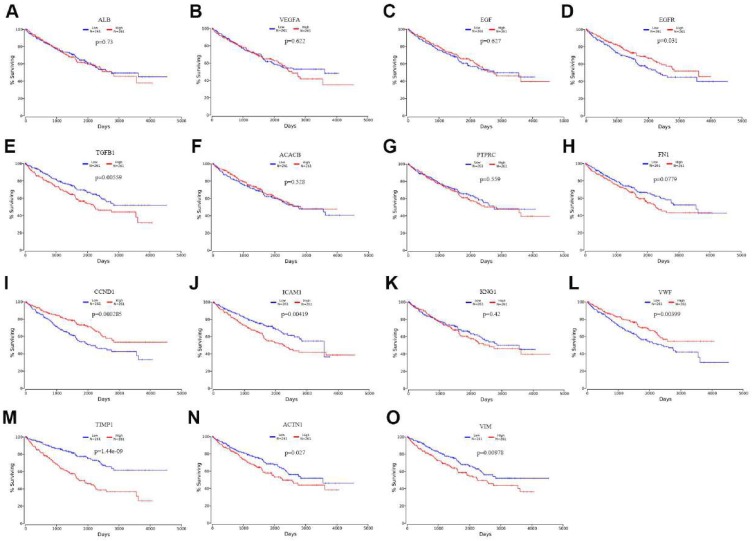
The overall survival analysis of top 15 hub genes in ccRCC: (A) ALB, (B) VEGFA, (C) EGF, (D) EGFR, (E) TGFB1, (F) ACACB, (G) PTPRC, (H) FN1, (I) CCND1, (J) ICAM1, (K) KNG1, (L) VWF, (M) TIMP1, (N) ACTN1, (O) VIM.

**Figure 4:**
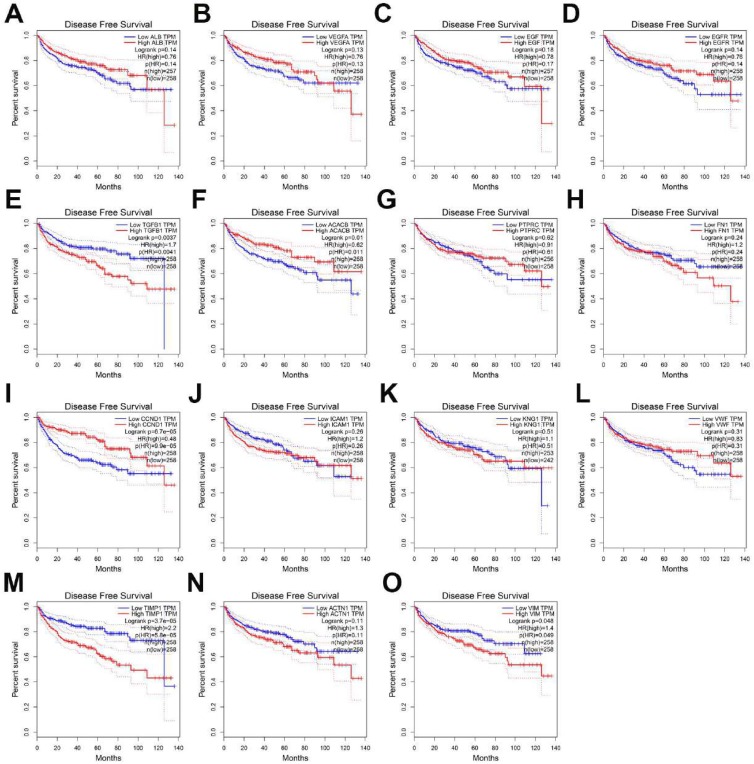
The disease-free survival analysis of top 15 hub genes in ccRCC: (A) ALB, (B) VEGFA, (C) EGF, (D) EGFR, (E) TGFB1, (F) ACACB, (G) PTPRC, (H) FN1, (I) CCND1, (J) ICAM1, (K) KNG1, (L) VWF, (M) TIMP1, (N) ACTN1, (O) VIM.

To evaluate the prognostic value of the top 15 hub genes, we performed a Kaplan meier curve analysis for overall survival (OS) at http://www.oncolnc.org/. As shown in Figure 3, the high expression of TGFB1 (logrank p=0.00559), ICAM1 (logrank p=0.00419), TIMP1 (logrank p=1.44e-09), ACTN1 (logrank p=0.027) and VIM (logrank p=0.00978) predicted a poor prognosis for ccRCC patients, while the high expression of EGFR (logrank p=0.031), CCND1 (logrank p=0.000285) and VMF (logrank p=0.00399) predicted a favorable prognosis for ccRCC patients. However, the expression levels of ALB (logrank p=0.73), VEGFA (logrank p=0.622), EGF (logrank p=0.627), ACACB (logrank p=0.528), PTPRC (logrank p=0.559), FN1 (logrank p=0.0779) and KNG1 (logrank p=0.42) were not associated with OS prognosis of ccRCC patients. Then, we evaluated the disease-free survival (DFS) prognosis of the top 15 hub genes at http://gepia.cancer-pku.cn/. As shown in Figure 4, the high expression of TGFB1 (logrank p=0.0037), TIMP1 (logrank p=3.7e-05), and VIM (logrank p=0.048) indicated a poor prognosis for ccRCC patients, while the high expression of CCND1 (logrank p=6.7e-05) indicated a favorable prognosis for ccRCC patients. However, the expression levels of the remaining hub genes were not correlated with disease-free survival (DFS) prognosis of ccRCC patients. From the above analysis, we found that the expression of CCND1, TGFB1, TIMP1 and VIM predicted a consistent overall survival and disease-free survival prognosis, in which high CCND1 expression predicted a good prognosis, while high expression of the other three genes predicted poor Prognosis. But these four hub genes were all highly expressed in ccRCC, so we selected TGFB1, TIMP1 and VIM as our research focus, which have great potential as prognosis biomarkers for ccRCC.

### The TCGA and HPA validation and the assessment of diagnostic value

**Figure 5:**
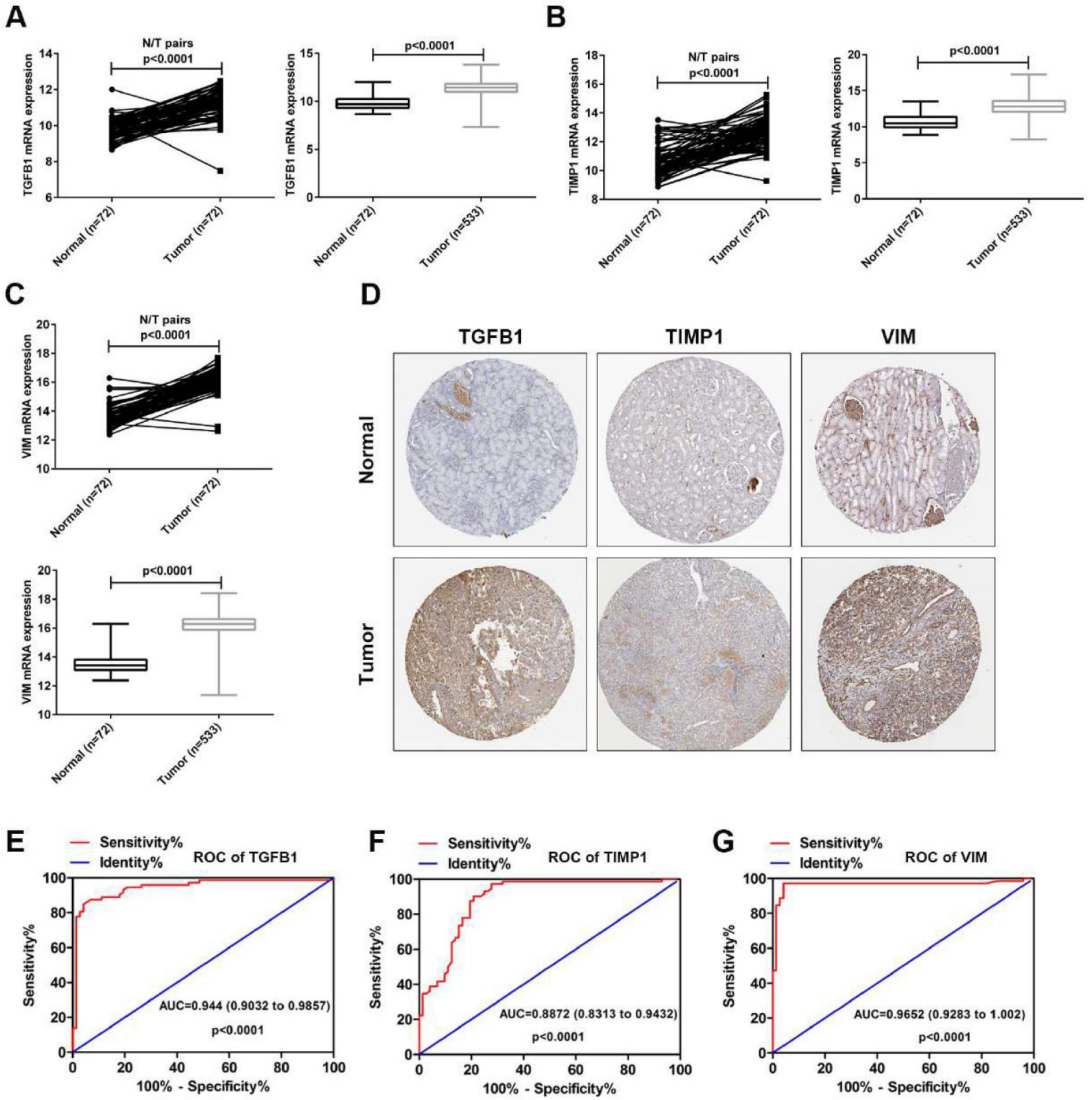
The expression levels and diagnostic value of TGFB1, TIMP1 and VIM in ccRCC. (A) The mRNA expression levels of TGFB1 in paired and unpaired ccRCC tissues from TCGA. (B) The mRNA expression levels of TIMP1 in paired and unpaired ccRCC tissues from TCGA. (C) The mRNA expression levels of VIM in paired and unpaired ccRCC tissues from TCGA. (D) The protein expression levels of TGFB1, TIMP1 and VIM in ccRCC from HPA. (E-G) The ROC curve analysis of TGFB1, TIMP1 and VIM in ccRCC tissues from TCGA.

To verify the accuracy of the GSE15641 dataset, the mRNA levels of TGFB1, TIMP1 and VIM were downloaded from the TCGA database. As shown in Fig. 5A-C, the mRNA levels of TGFB1, TIMP1 and VIM were significantly upregulated in the paired and unpaired ccRCC tissues compared to the normal tissues. Then, we analyzed the protein expression levels of these three hub genes using The Human Protein Atlas (HPA, https://www.proteinatlas.org/). As shown in Fig. 5D, the protein levels of TGFB1, TIMP1 and VIM were obviously upregulated in the ccRCC tissues compared to the normal tissues. Finally, we used TGFB1, TIMP1 and VIM mRNA levels from TCGA to evaluate their diagnostic value. ROC curve analysis showed that TGFB1, TIMP1 and VIM expression levels have potential diagnostic value for ccRCC patients (Fig. 5E-G). These results confirm the high expression of TGFB1, TIMP1 and VIM in ccRCC and their diagnostic value for ccRCC.

### The relationship between the expression of hub genes and ccRCC stage

**Figure 6:**
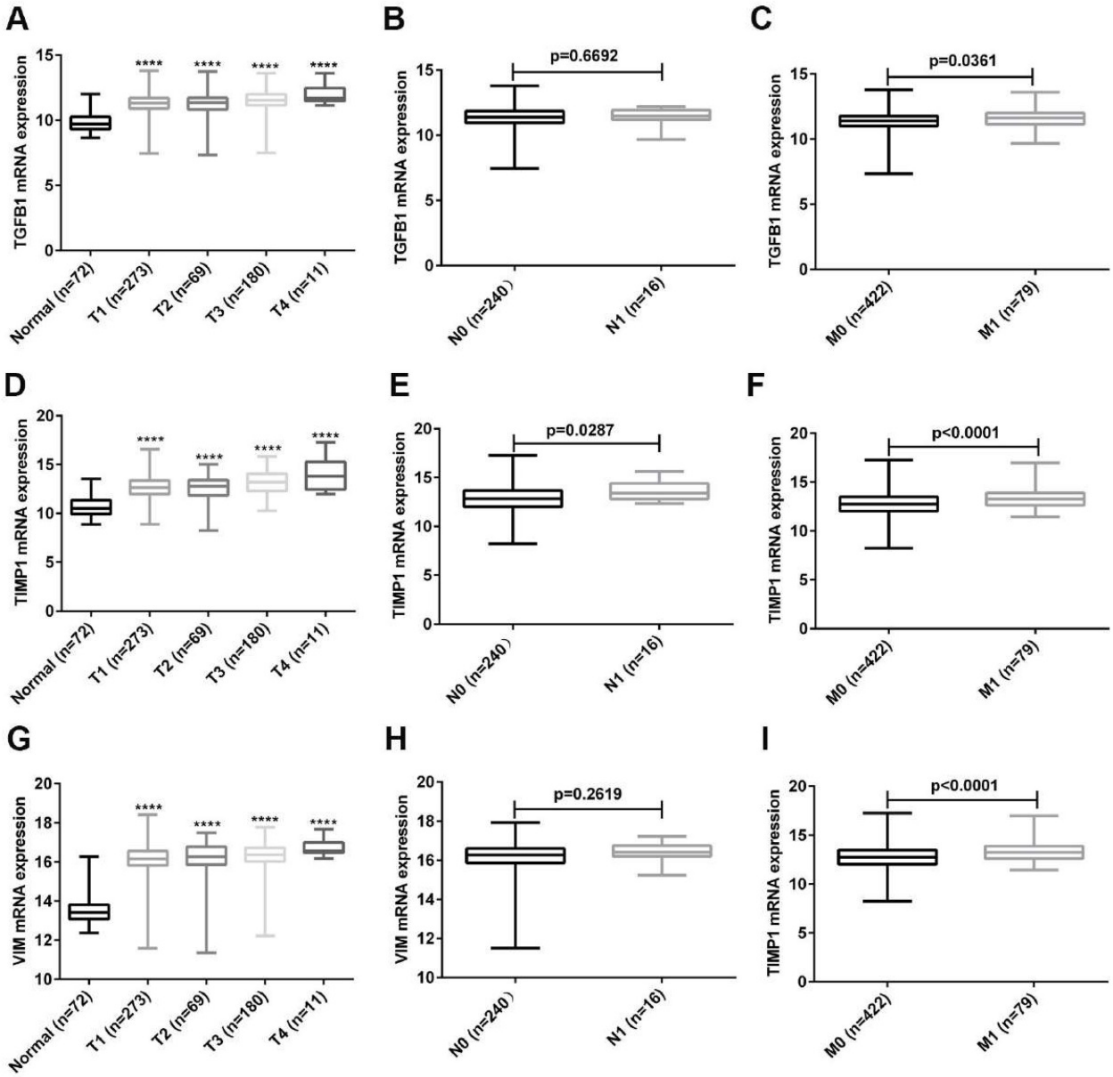
The relationship between expression of TGFB1, TIMP1 and VIM and TNM stage. (A-C) The relationship between TGFB1 expression and TNM stage. (D-F) The relationship between TIMP1 expression and TNM stage. (G-I) The relationship between VIM expression and TNM stage. (****, P<0.0001, compared with the normal tissues).

To further explore the clinical value of these three hub genes, we analyzed the relationship between their expression and TNM stage. As shown in Fig. 6A, the expression of TGFB1 was up-regulated as the T stage increased, and the expression of TGFB1 in M1 patients was higher than that of M0 patients (Fig. 6C). Unfortunately, the expression of TGFB1 was not related to the N stage of ccRCC (Fig. 6B). Next, we analyzed the relationship between TIMP1, VIM expression and TNM stage. The results obtained were consistent with the expression analysis of TGFB1 (Fig. 6D-I). Unexpectedly, the expression of TIMP1 was positively correlated with N stage (Fig. 6E).

### The Gene Set Enrichment Analysis of the hub genes

**Figure 7:**
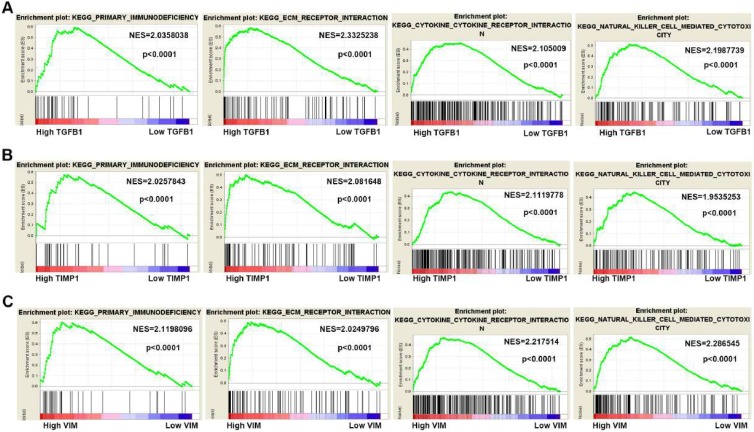
Gene Set Enrichment Analysis of hub genes in ccRCC. (A-C) High TGFB1, TIMP1 and VIM expression co-enriched gene sets in ccRCC are listed, including "primary immunodeficiency", "ECM receptor interaction", "cytokine receptor interaction" and "natural killer cell-mediated cytotoxicity". (NES means Normalized Enrichment Score)

To gain insight into the biological functions of hub genes, GSEA analysis was manipulated to predict the biological processes involved by hub genes. According to the NES (normalized enrichment score) > 2, FDR (the false discovery rate) < 0.05 and the nominal P value < 0.05, high TGFB1, TIMP1 and VIM expression were mainly enriched in “primary immunodeficiency”, “ECM receptor interaction”, “cytokine receptor interaction” and “natural killer cell-mediated cytotoxicity” pathways (Fig. 7A-C).

## Discussion

The ccRCC is the most common malignant tumor that occurs in the kidney and is characterized by strong metastatic ability and poor prognosis. In recent years, despite the improvement of surgical techniques and advances in molecular targeted therapy and immunotherapy, the prognosis of ccRCC is still not ideal. Therefore, sensitive and specific biomarkers for early diagnosis of ccRCC are urgently needed to be identified, aiming to implement early treatment and improve patient outcomes. High-throughput sequencing studies can facilitate deep exploration of the mechanisms contributing to ccRCC progression 13. Thus, our study performed a deep analysis of the microarray expression profile from GSE15641 dataset, which included 23 normal kidney tissues and 32 ccRCC tissues. A total of 598 DEGs were screened out including 406 up-regulated genes and 192 down-regulated genes. Moreover, we identified the top 15 hub genes from the DEGs using the PPI network and Cytoscape software.

To deeply explore the biological pathways and functions involved by these DEGs, we performed GO and KEGG analysis. To find the key genes for ccRCC progression from the numerous DEGs, we identified the top 15 hub genes through the PPI network and Cytoscape. The top 15 hub genes include ALB, VEGFA, EGF, EGFR, TGFB1, ACACB, PTPRC, FN1, CCND1, ICAM1, KNG1, VWF, TIMP1, ACTN1 and VIM. We used Kaplan-Meier curves to analyze the association of top 15 hub genes expression with OS and DFS in ccRCC. The results showed that only three hub genes (TGFB1, TIMP1 and VIM) were related to the OS and DFS of ccRCC. Therefore, we regard these three hub genes as the focus of our research.

To confirm the expression of these three hub genes in the GSE15641 dataset, the TCGA database was used to verify the expression of their mRNA levels and HPA online tool was used to validate the expression of their protein levels. The results obtained by TCGA and HPA verification are consistent with the results of GSE15641 dataset. We then evaluated the diagnostic value of these three hub genes using the ROC curve, indicating that they have potential diagnostic value for ccRCC. These results indicate that these three hub genes may be new diagnostic and prognostic biomarkers for ccRCC.

The gene TGFB1 encodes a secreted ligand of the TGF-beta (transforming growth factor-beta) superfamily of proteins. TGFB1 protein can induce epithelial-to -mesenchymal transition (EMT) and cell migration in various cell types 14, 15. Also TGFB1 mediates SMAD2/3 activation by inducing its phosphorylation and subsequent translocation to the nucleus 14. Moreover, TGFB1 may serve as a prognostic biomarker for hepatocellular carcinoma 16. Integrating our ROC curve analysis and survival analysis, we speculated that TGFB1 might be a potential indicator for the diagnosis and prognosis of ccRCC.

The TIMP1 gene belongs to the TIMP gene family. The proteins encoded by this gene family are natural inhibitors of the matrix metalloproteinases (MMPs), a group of peptidases involved in degradation of the extracellular matrix. In addition to its inhibitory role against most known MMPs, the TIMP1 protein is able to promote cell proliferation in a wide range of cell types, and may also have an anti-apoptotic function 17-19. The inhibition of TIMP1 expression reduced proliferation and metastasis but increased apoptosis by activating TIMP1 mediated FAK-PI3K/AKT and MAPK pathway 20. TIMP1, serve as a hub gene of gastric cancer, may be a potential prognostic biomarker for gastric cancer 21. Our study found that TIMP1 is up-regulated in ccRCC and has potential as a diagnostic and prognostic biomarker.

The VIM gene encodes a type III intermediate filament protein. Intermediate filaments, along with microtubules and actin microfilaments, make up the cytoskeleton. The VIM protein is responsible for maintaining cell shape and integrity of the cytoplasm, and stabilizing cytoskeletal interactions. Moreover, as a mesenchymal cell protein, VIM is a key factor in the EMT process. Previous study has reported that FSTL1 binds to VIM and facilitates colorectal cancer metastasis through activating the focal adhesion signalling pathway 22. VIM has also been reported as one of the most promising biomarkers for early diagnosis and prognosis prediction of tongue squamous cell carcinoma 23. Our study found that VIM is highly expressed in ccRCC and has diagnostic and prognostic value for ccRCC.

Through a series of bioinformatics analysis, we will make a summary here. In conclusion, 598 DEGs were screened out from the GSE15641 dataset, which may contain hub genes contributing to the pathogenesis of ccRCC. Through our bioinformatics analysis, three of the top 15 hub genes including TGFB1, TIMP1 and VIM might contribute to the progression of ccRCC, which could serve as novel diagnostic and prognostic biomarkers and therapeutic targets for ccRCC.

## Figures and Tables

**Table 1:** The top 15 hub genes with a higher degree of connectivity in GSE15641 dataset

Gene name	Degree of connectivity	Adjusted P value	Gene Expression
ALB	100	7.57E-17	Downregulated
VEGFA	67	1.74E-13	Upregulated
EGF	65	2.23E-19	Downregulated
EGFR	60	4.55E-11	Upregulated
TGFB1	52	5.23E-09	Upregulated
ACACB	45	4.90E-26	Downregulated
PTPRC	41	1.08E-13	Upregulated
FN1	39	5.05E-21	Upregulated
CCND1	38	1.47E-16	Upregulated
ICAM1	37	4.42E-33	Upregulated
KNG1	35	2.41E-29	Downregulated
VWF	31	4.86E-16	Upregulated
TIMP1	30	1.62E-22	Upregulated
ACTN1	30	4.93E-21	Upregulated
VIM	30	5.25E-17	Upregulated

**Table 2:** The GO analysis of differentially expressed genes in the ccRCC

Gene expression	Category	Term	Count	%	PValue	FDR
**Upregulated**	GOTERM_CC_DIRECT	GO:0031012~extracellular matrix	31	8.93	2.21E-14	3.04E-11
	GOTERM_CC_DIRECT	GO:0005615~extracellular space	61	17.57	6.12E-11	8.41E-08
	GOTERM_CC_DIRECT	GO:0005581~collagen trimer	15	4.32	1.09E-09	1.50E-06
	GOTERM_CC_DIRECT	GO:0005576~extracellular region	65	18.73	1.39E-09	1.92E-06
	GOTERM_CC_DIRECT	GO:0009986~cell surface	33	9.51	4.67E-09	6.42E-06
	GOTERM_BP_DIRECT	GO:0030198~extracellular matrix organization	26	7.49	5.52E-14	9.56E-11
	GOTERM_BP_DIRECT	GO:0001666~response to hypoxia	22	6.34	1.42E-11	2.46E-08
	GOTERM_BP_DIRECT	GO:0007155~cell adhesion	34	9.79	5.95E-11	1.03E-07
	GOTERM_BP_DIRECT	GO:0002576~platelet degranulation	14	4.03	7.88E-08	1.36E-04
	GOTERM_BP_DIRECT	GO:0030199~collagen fibril organization	9	2.59	5.86E-07	0.001014072
	GOTERM_MF_DIRECT	GO:0005178~integrin binding	14	4.03	7.23E-08	1.07E-04
	GOTERM_MF_DIRECT	GO:0005201~extracellular matrix structural constituent	10	2.88	3.84E-06	0.005662561
	GOTERM_MF_DIRECT	GO:0005515~protein binding	203	58.50	4.39E-06	0.006464779
	GOTERM_MF_DIRECT	GO:0042605~peptide antigen binding	7	2.01	1.03E-05	0.015247132
	GOTERM_MF_DIRECT	GO:0042803~protein homodimerization activity	32	9.22	1.59E-05	0.023366428
**Downregulated**	GOTERM_CC_DIRECT	GO:0070062~extracellular exosome	41	23.83	6.28E-04	0.793504017
	GOTERM_CC_DIRECT	GO:0005615~extracellular space	22	12.79	0.005456219	6.698549756
	GOTERM_CC_DIRECT	GO:0031093~platelet alpha granule lumen	4	2.32	0.011661381	13.81292879
	GOTERM_CC_DIRECT	GO:0005576~extracellular region	22	12.79	0.034131924	35.60273157
	GOTERM_CC_DIRECT	GO:0016324~apical plasma membrane	7	4.06	0.039386897	39.90451163
	GOTERM_BP_DIRECT	GO:0042493~response to drug	15	8.72	8.17E-07	0.001307793
	GOTERM_BP_DIRECT	GO:0034765~regulation of ion transmembrane transport	6	3.48	0.003572931	5.565651774
	GOTERM_BP_DIRECT	GO:0008285~negative regulation of cell proliferation	11	6.39	0.003600942	5.608115289
	GOTERM_BP_DIRECT	GO:0007568~aging	7	4.06	0.004178027	6.478970288
	GOTERM_BP_DIRECT	GO:0007588~excretion	4	2.32	0.004677244	7.226235366
	GOTERM_MF_DIRECT	GO:0042802~identical protein binding	18	10.46	5.70E-04	0.774996196
	GOTERM_MF_DIRECT	GO:0005215~transporter activity	7	4.06	0.011237989	14.28183863
	GOTERM_MF_DIRECT	GO:0042813~Wnt-activated receptor activity	3	1.74	0.017364869	21.24766214
	GOTERM_MF_DIRECT	GO:0046934~phosphatidylinositol-4,5-bisphosphate 3-kinase activity	4	2.32	0.019675974	23.73609635
	GOTERM_MF_DIRECT	GO:0017147~Wnt-protein binding	3	1.74	0.033119697	36.82480288

**Table 3:** KEGG pathway analysis of differentially expressed genes in the ccRCC

Category	Term	Count	Generatio (%)	PValue
**Up-regulated DEGs**	hsa04510:Focal adhesion	26	7.49	1.05E-10
	hsa04145:Phagosome	21	6.05	2.41E-09
	hsa04512:ECM-receptor interaction	16	4.61	5.74E-09
	hsa05146:Amoebiasis	16	4.61	9.13E-08
	hsa05416:Viral myocarditis	12	3.45	2.01E-07
**Down-regulated DEGs**	hsa00010:Glycolysis / Gluconeogenesis	6	3.48	0.001069
	hsa00350:Tyrosine metabolism	4	2.32	0.007754
	hsa01100:Metabolic pathways	23	13.37	0.022939
	hsa04922:Glucagon signaling pathway	5	2.90	0.028494
	hsa00640:Propanoate metabolism	3	1.74	0.04214
